# Resistance exercise increases active MMP and *β*1‐integrin protein expression in skeletal muscle

**DOI:** 10.14814/phy2.12212

**Published:** 2014-11-20

**Authors:** Riki Ogasawara, Koichi Nakazato, Koji Sato, Marni D. Boppart, Satoshi Fujita

**Affiliations:** 1 Department of Life Sciences, The University of Tokyo, Tokyo, Japan; 2 Faculty of Sport and Health Science, Ritsumeikan University, Shiga, Japan; 3 Graduate School of Health and Sport Science, Nippon Sport Science University, Tokyo, Japan; 4 Department of Kinesiology and Community Health, Beckman Institute for Advanced Science and Technology, University of Illinois, Urbana, Illinois

**Keywords:** Exercise, gelatinases, integrin, mTOR, muscle hypertrophy

## Abstract

Recent studies indicate that matrix metalloproteinases (MMPs) and critical linkage proteins in the extracellular matrix (ECM) regulate skeletal muscle mass, although the effects of resistance training (RT) on protein expression and activity are unclear. Thus, the purpose of the present study was to investigate the effects of RT on MMP activity and expression of ECM‐related proteins. Ten male Sprague–Dawley rats were randomly assigned to 1 bout (1B) or 18 bouts (18B) of electrical stimulation. The right gastrocnemius muscle was isometrically contracted via percutaneous electrical stimulation (five sets of 5 sec stimulation × five contractions/set with 5 sec interval between contractions and 3 min rest between sets) once (1B) or every other day for 5 weeks (18B). The left leg served as a control. Activity of MMP‐2 and MMP‐9, determined via gelatin zymography, was increased (*P *<**0.05) immediately after 1B. However, MMP activation was not evident following 18B. No changes in collagen IV, laminin *α*2, *α*7‐integrin, or ILK protein expression were detected immediately following 1B or 18B. However, *β*1‐integrin protein expression was significantly increased (*P *<**0.05) with 18B. Our results suggest that resistance exercise activates MMPs during the initial phase of RT but this response is attenuated with continuation of RT.

## Abstract (precis)

Recent studies indicate that matrix metalloproteinases (MMPs) and critical linkage proteins in the extracellular matrix (ECM) regulate skeletal muscle mass, although the effects of resistance training (RT) on protein expression and activity are unclear. Our results suggest that resistance exercise activates MMPs during the initial phase of RT but this response is attenuated with continuation of RT.

## Introduction

Resistance training is generally acknowledged as an effective tool to increase skeletal muscle mass. An acute bout of resistance training can activate mTORC1 signaling, an early event considered to be responsible for skeletal muscle hypertrophy with continuation of training (Mayhew et al. 2009, 2011; Terzis et al. 2010). We and others have reported that intracellular signaling responses to resistance exercise are attenuated with chronic resistance training (Ogasawara et al. 2013a; Nader et al. 2014), suggesting that the primary mechanisms for activation of mTORC1 signaling are similarly altered. Therefore, models of resistance training provide the opportunity to elucidate putative upstream regulators of mTORC1 signaling. Identifying the upstream molecular targets responsible for the muscle anabolic response to resistance exercise is a prerequisite for the development of exercise and/or nutritional interventions.

Previous studies have indicated that the two primary extracellular matrix (ECM) linkage complexes necessary for muscle integrity, dystrophin–dystroglycan (Spence et al. 2004; Xiong et al. 2009; Leonoudakis et al. 2010) and integrin (Boppart et al. 2006; Zou et al. 2011; Marshall et al. 2012), and basal lamina components such as type IV collagen and laminin (Grounds et al. 2005; Gillies and Lieber 2011) significantly influence hypertrophic signaling, including mTORC1 signaling, and regulation of muscle mass. In many cases, changes in skeletal muscle mass and function as a result of aging, altered muscle activity, and skeletal muscle disease such as muscle dystrophy occur with concomitant loss of ECM structure (Urso et al. 2006; Kragstrup et al. 2011; Ramaswamy et al. 2011), indicating that changes in membrane‐stabilizing proteins have the ability to inhibit and potentiate cellular signaling with resistance exercise and training. However, changes in ECM‐related proteins in response to resistance training are relatively unknown.

Matrix metalloproteinases (MMPs) are a family of zinc‐ and calcium‐dependent proteolytic enzymes responsible for extracellular matrix (ECM) degradation (Carmeli et al. 2004; Alameddine 2012). MMP‐2 and MMP‐9 (gelatinase A and B, respectively) are proteolytic enzymes that target components of the basal lamina, particularly type IV collagen (Carmeli et al. 2004; Alameddine 2012). Recent studies have demonstrated that elevated levels of active MMP‐9 result in upregulation of Akt/mTOR signaling pathway and muscle hypertrophy and that MMP‐9 deficiency causes muscle atrophy and changes in muscle fiber type composition (Dahiya et al. 2011; Mehan et al. 2011), suggesting that early changes in MMP‐9 activity are associated with muscle protein metabolism and muscle mass regulation. However, the effects of chronic resistance exercise on MMP‐9 and MMP‐2 activity are unknown.

Thus, the purpose of this study was to evaluate MMP activation and ECM protein expression immediately following both an acute bout of resistance exercise and chronic resistance training. We hypothesized that MMP activation would be temporally altered with an acute bout of exercise and downregulated with training, whereas ECM protein expression would be increased with resistance training.

## Methods

### Animals

Ten Sprague–Dawley rats (male, aged 10 weeks) were obtained from CLEA Japan (Tokyo, Japan). All animals were housed individually in an environment maintained at 22–24°C with a 12‐h light‐dark cycle and were allowed food and water ad libitum. Rats were randomly assigned to 1 bout (1B) or 18 bouts (18B) of electrical stimulation. This study was approved by the Ethics Committee for Animal Experiments at Ritsumeikan University.

### Electrical simulation protocol

Methods for activation of muscle contraction were performed as previously reported (Ogasawara et al. 2013a). Briefly, under isoflurane anesthesia, the hair was shaved off the right lower leg of each rat, and the shaved legs were cleaned with alcohol wipes. Rats were then positioned with their right foot on the footplate of the training device (the ankle joint angle was positioned at 90°) in the prone posture. The gastrocnemius muscle was stimulated percutaneously with electrodes (Vitrode V; Ag/AgCl; Nihon Kohden, Tokyo, Japan), which were cut to 10 × 5 mm sections and connected to an electric stimulator and an isolator (SS‐104J; Nihon Kohden, Tokyo, Japan).

Rats were acclimatized for 1 week, and the right gastrocnemius muscle was then isometrically contracted (five sets of stimulation for 5 sec × five contraction, with a 5‐sec interval between contractions and 3 min rest intervals between sets) every other day. The left gastrocnemius muscle served as an internal control. The voltage (~30 V) and stimulation frequency (60 Hz) were adjusted to produce maximal isometric tension. In the previous study, we demonstrated that this training protocol induced significant increases in muscle size and strength (Ogasawara et al. 2013a). Immediately after the last exercise session, the rats were anesthetized and exsanguinated. Right and left gastrocnemius were removed immediately after death. The tissues were rapidly frozen in liquid N_2_ and stored at −80°C until use.

### Western blotting analysis

Muscle samples were homogenized with a Polytron homogenizer in a homogenization buffer containing 20 mmol/L Tris‐HCl pH 7.5, 1% NP40, 1% sodium deoxycholate, 1 mmol/L EDTA, 1 mmol/L EGTA, 150 mmol/L NaCl, and protease and phosphatase inhibitor cocktail (Thermo Fisher Scientific, Waltham, MA). Homogenates were centrifuged at 10,000 *g* for 10 min at 4°C. The supernatant was removed, and the protein concentration for each sample was determined using a protein concentration determination kit (Protein Assay Rapid kit; WAKO, Osaka, Japan). The samples were diluted in 3× sample buffer and boiled at 95°C for 5 min. Using 5–20% SDS‐polyacrylamide gradient gels, 50 *μ*g of protein was separated by electrophoresis and subsequently transferred to polyvinylidene difluoride membranes. After transfer, the membranes were blocked with 5% powdered milk for 1 h at room temperature and incubated overnight at 4°C with primary antibodies targeting Collagen IV (cat# ab6586; Abcam, Cambridge, MA), laminin *α*2 (cat# MAB1922; Millipore, Billerica, MA), *α*‐dystroglycan (cat# 05‐593; Millipore), *β*‐dystroglycan (cat# sc‐16165; Santa Cruz Biotechnology, Santa Cruz, CA), Integrin *α*7B (a gift from M.D. Boppart), Integrin *β*1D (cat# MAB1900; Millipore), and integrin‐linked kinase 1 (ILK; cat# 3862; Cell Signaling Technology, Danvers, MA). The membranes were then incubated for 1 h at room temperature with appropriate secondary antibodies. Chemiluminescent reagents (ECL Plus; GE Healthcare, Milwaukee, WI) were used for detection of protein bands. The bands were imaged using a chemiluminescence detector (ImageQuant LAS 4000; GE Healthcare). After the scan, the membranes were stained with Coomassie Brilliant Blue (CBB) to verify and normalize protein loading (Welinder and Ekblad 2011). The band intensities were quantified using ImageJ 1.46 software (National Institutes of Health (NIH); Bethesda, MD).

### Myosin heavy chain (MHC) composition analysis

Myosin heavy chain composition was analyzed according to a previously described procedure (Mizunoya et al. 2008). Briefly, muscle samples were homogenized in an SDS solution containing 10% w/v SDS, 40 mmol/L DTT, 5 mmol/L EDTA, 0.1 mol/L Tris‐HCL buffer pH 8.0, and protease inhibitor cocktail (Thermo Fisher Scientific). After centrifugation, determination of protein concentration, and boiling with sample buffer, the proteins were separated by electrophoresis (8% separating gel and 4% stacking gel; glycerol 35% w/v in separating gel and 30% w/v in stacking gel). Electrophoresis was performed at a constant voltage of 140 V for 22 h at 4°C, and the gels were then stained using a silver staining kit (Silver stain KANTO III; Kanto Chemical, Tokyo, Japan).

### Zymography

The enzymatic activity of MMP‐2 and MMP‐9 in skeletal muscle was measured using a gelatin zymography kit (Primary Cell Co., Ltd., Sapporo, Japan) according to the manufacturer's protocol. Briefly, muscle samples were homogenized with a Polytron homogenizer in a homogenization buffer similar to that used for western blotting, except that it did not contain EDTA. The homogenates were centrifuged, the supernatant was removed, and the protein concentration was determined. The samples were diluted in sample buffer and incubated at room temperature for 15 min. Using SDS‐polyacrylamide gels containing gelatin, 50 *μ*g of protein was separated by electrophoresis. The gels were developed according to the manufacturer's instructions and stained with CBB; band intensities were then quantified using ImageJ 1.46 software (NIH).

### Statistical analysis

Changes in protein expression were compared by two‐way ANOVA (stimulation × training status). Post hoc analyses were performed using *t*‐tests with the Benjamini and Hochberg false discovery rate correction for multiple comparisons. All values were expressed as the mean ± SEM. Significance was accepted at *P *<**0.05.

## Results

### MHC composition

The MHC composition in skeletal muscle following repeated muscle contraction (18B) compared to a single event (1B) is presented in Table 1. Following chronic muscle contraction, we observed a significant increase in the relative proportion of type IIx MHC, whereas there was a significant decline in the relative proportion of type IIb MHC. The relative proportion of type I MHC and type IIa MHC did not change in the present study.

**Table 1.** Myosin heavy chain composition before and after chronic resistance training.

	1B	18B
Type I, %	9.1 ± 0.4	9.6 ± 0.9
Type IIa, %	16.0 ± 0.6	16.7 ± 0.4
Type IIx, %	32.8 ± 1.0	42.0 ± 1.2*
Type IIb, %	42.1 ± 0.8	31.7 ± 0.7*

Values are means ± SEM. 1B, 1 bout group; 18B, 18 bouts group.

* *P *< 0.05 versus 1B.

### MMPs

**Figure 1.**
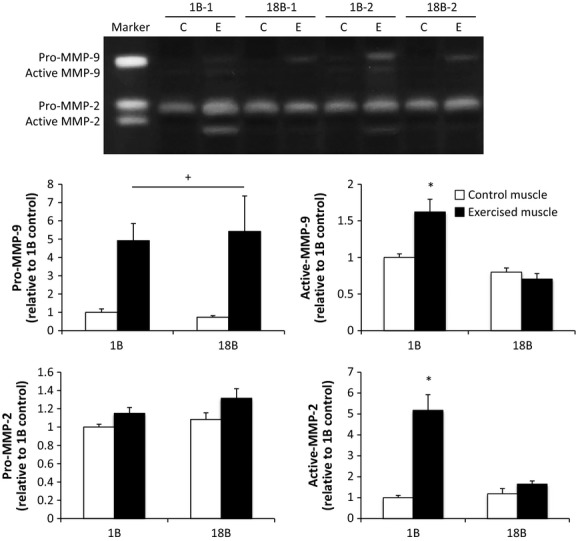
Effect of acute and chronic resistance exercise on matrix metalloproteinase (MMP) activity. Values are the mean ± SEM. 1B, 1 bout group; 18B, 18 bouts group; C, control muscle; E, exercised muscle. *n* = 2 for each condition. *P <**0.05 versus control muscle; +, *P *<**0.05 main effect of exercise (no interaction).

A single bout of electrical stimulation elevated the level of active MMP‐2, pro‐MMP‐9, and active MMP‐9; a similar increase in pro‐MMP‐9 was observed with 18B (Fig. 1). However, repeated bouts of muscle contraction blunted the level of active MMP‐2 and MMP‐9 with 18B.

### Collagen and laminin

**Figure 2.**
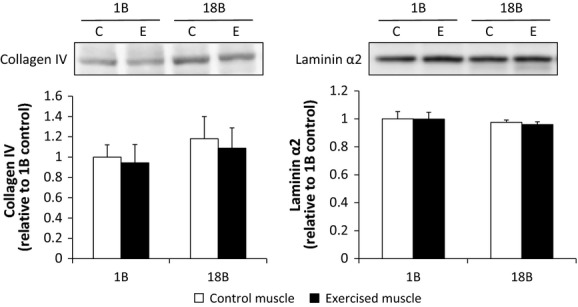
Effect of acute and chronic resistance exercise on basal lamina proteins. Values are the mean ± SEM. 1B, 1 bout group; 18B, 18 bouts group; C, control muscle; E, exercised muscle.

There was no significant effect of acute or chronic muscle contraction on collagen IV and laminin *α*2 protein expression (Fig. 2).

### Dystroglycans

**Figure 3.**
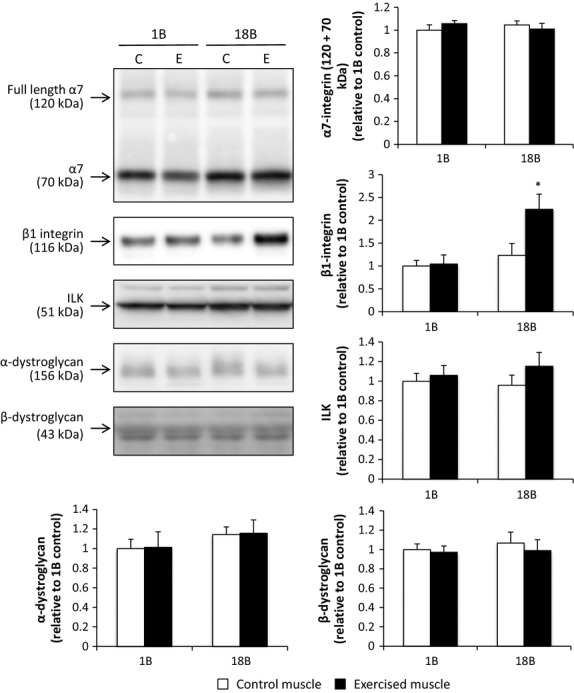
Effect of acute and chronic resistance exercise on integrin‐related proteins and dystroglycans. Values are the mean ± SEM. 1B, 1 bout group; 18B, 18 bouts group; C, control muscle; E, exercised muscle. *P <**0.05 versus control muscle.

No significant change in *α*‐ and *β*‐dystroglycan protein expression was observed after acute or chronic muscle contraction (Fig. 3).

### Integrin subunits

There was no significant effect of acute muscle contraction on *α*7‐, and *β*1‐integrin protein expression, but *β*1‐integrin protein was significantly increased 2.X‐fold after chronic muscle contraction (Fig. 3).

## Discussion

In the present study, we investigated changes in MMP activity and ECM protein expression in skeletal muscle in response to chronic resistance training simulated by repeated electrical stimulation. Our main finding is that the predominant gelatinases, MMP‐2 and MMP‐9, were activated immediately following an acute bout of contraction; however, repeated bouts of contraction blunted this effect. In contrast, pro‐MMP‐9 activity was still activated with training. With the exception of the *β*1‐integrin subunit, no changes in ECM protein expression were observed in muscle with repeated bouts of electrical stimulation.

We evaluated the levels of pro‐ and active gelatinase by gelatin zymography and observed elevated levels of pro‐MMP‐9, active MMP‐9, and active MMP‐2 following acute resistance exercise. Several other studies have evaluated gelatinase activity or protein expression following a single bout of exercise (Carmeli et al. 2005; Rullman et al. 2007, 2009). Rullman et al. (Rullman et al. 2009) reported that MMP‐9 activity, but not MMP‐2 activity, was enhanced in human skeletal muscle 2 h after 45 min of endurance exercise. The fact that gelatinase activity or expression is largely influenced by mechanical stimulation in capillary and tendon tissue (Koskinen et al. 2004; Milkiewicz and Haas 2005; Heinemeier et al. 2007; Milkiewicz et al. 2007), suggests that strain associated with contraction may serve as a strong regulator of gelatinase activity observed post exercise. Thus, the high degree of mechanical strain associated with resistance exercise may account for discrepancies in MMP activities previously reported with endurance exercise.

In this study, we also investigated the effect of chronic muscle contraction on gelatinase activity. Although the levels of pro‐MMP‐9, active MMP‐9, and active MMP‐2 were elevated after acute muscle contraction and before chronic training, increased level after chronic training was observed for pro‐MMP‐9 only. Because data on the baseline levels of gelatinases after chronic resistance training are unavailable, we could not determine whether the elevated level of pro‐MMP‐9 resulted from acute or chronic muscle contraction. However, pretraining baseline levels of active MMP‐2 and MMP‐9 were very low, which suggest that active MMP‐2 and MMP‐9 responses to exercise stimulation may be desensitized in chronically trained muscle even during maximal contraction.

In contrast to our study, Deus et al. (2012) showed that pro‐MMP‐2 and MMP‐2 activity was elevated after 8 weeks of resistance training (three times per week), although it is unclear whether acute exercise or chronic exercise training contributed to these elevated activities, as no information was provided concerning the effect of initial exercise on activity. One possible reason for the observed difference between our findings and those of Deus et al. may be the difference in the skeletal muscle targeted. In this study, we used the gastrocnemius (GST) muscle, whereas Deus et al. used the tibialis anterior (TA) muscle. It is known that the TA muscle consists predominantly of type IIb fibers (80%; Staron et al. 1999); however, the GST muscle consists of mainly type IIx and IIb fibers (IIx: 33%, IIb: 42%, respectively), suggesting that the GST muscle is more oxidative than the TA muscle. A previous study demonstrated that gelatinase expression is altered to a greater degree in glycolytic muscle fibers, although that study did not measure gelatinase activity (Carmeli et al. 2005). Therefore, gelatinase responsiveness to muscle contraction may be higher in fast‐twitch muscle fibers than in slow‐twitch muscle fibers.

Overexpression of the active version of MMP‐9 can result in reduced collagen IV protein in skeletal muscle (Dahiya et al. 2011). In the present study, resistance training did not alter total collagen IV protein content, despite increased gelatinase activity. The fact that collagen IV mRNA is increased following an acute bouts of exercise (Han et al. 1999; Koskinen et al. 2001), suggests that chronic resistance training may allow for collagen IV protein turnover, as opposed to accretion, and structural remodeling of the basal lamina to accommodate fiber growth.

Previous studies have reported increased *α*7 integrin subunit mRNA and protein expression in skeletal muscle 3 and 24 h following a single bout of eccentric exercise, respectively (Boppart et al. 2006, 2008). Thus, we hypothesized that chronic resistance training would increase expression of the critical linkage proteins necessary for skeletal muscle integrity. Chronic resistance training increased *β*1‐integrin protein, whereas protein expression of its major binding partner, *α*7‐integrin, remained unchanged. The fact that *α*7‐integrin subunit protein expression did not increase was unexpected. A previous study reported that overexpression of *β*1‐integrin results in increased *α*7‐integrin levels (Liu et al. 2012). In contrast, although overexpression of FAK, which plays an important role in integrin‐mediated signal transduction, increased *β*1‐integrin levels, *α*7‐integrin levels remained unchanged (Klossner et al. 2013). The reason for this discrepancy is unclear. However, the former result was obtained in 4 weeks after transfection, whereas the latter result was observed only 7 days after transfection. Therefore, it is possible that longer term *β*1‐integrin elevation results in increased *α*7‐integrin levels. Another possibility is that whether the *α*7‐integrin protein at the muscle membrane is a target for proteolytic cleavage and degradation post exercise is not known, but possible, given the potential for ECM turnover with resistance training.

We recently demonstrated that repeated application of an electrical stimulus can rapidly increase mTORC1 signaling and muscle growth which progressively decreases with time (Ogasawara et al. 2013a,b; Nader et al. 2014). From the present findings, it is difficult to associate the changes in gelatinase activation response and *β*1 integrin expression with a change in the muscle hypertrophic response. However, recent studies have shown that chronic MMP‐9 activation or inactivation alters muscle mass (hypertrophy or atrophy) and muscle fiber type (Dahiya et al. 2011; Mehan et al. 2011). Together, the results of the present and previous studies suggest that gelatinase activation in response to exercise may be greater after resistance exercise than after endurance exercise. In addition, the attenuated activation response of gelatinases to muscle contraction after chronic muscle contraction is similar to the muscle hypertrophic adaptation of muscle anabolism (i.e., resistance exercise‐induced mTOR activation) and subsequent hypertrophy that rapidly occur during the early phases of training and become progressively slower with time (Ogasawara et al. 2013a,b). Thus, although a causal relationship could not be determined in this study, activation of gelatinases in response to exercise may be associated with exercise‐induced muscle protein anabolism and hypertrophy. Similarly, *α*7*β*1 integrin is considered to negatively regulate mechanotransduction induced by exercise (Boppart et al. 2006), indicating that chronic resistance training‐induced changes in integrin expression affect the anabolic response of the muscle to an acute bout of resistance exercise. Although the functional role of MMPs and *β*1 integrin in exercise‐induced muscle protein anabolism is currently unclear, future studies should elucidate the functional role of exercise‐induced gelatinase activation and *β*1 integrin expression, and clarify the adaptive mechanisms of skeletal muscle in response to chronic muscle contraction.

## Conflict of Interest

The authors declare no conflicts of interest.
